# Whole-genome sequencing of *Mycobacterium tuberculosis* sublineage L1.1.1.7 from Chiangrai, Thailand

**DOI:** 10.1128/mra.00663-24

**Published:** 2024-09-23

**Authors:** Kanin Nuttavuthisit, Wuthiwat Ruangchai, Thavin Bodharamik, Waritta Sawaengdee, Surakameth Mahasirimongkol, Prasit Palittapongarnpim

**Affiliations:** 1 Shrewsbury International School, Bangkok, Thailand; 2 Emeritus Professor Pornchai Matangkasombut Center for Microbial Genomics, Department of Microbiology, Faculty of Science, Mahidol University, Bangkok, Thailand; 3 Medical Life Science Institute, Department of Medical Sciences, Ministry of Public Health, Nonthaburi, Thailand; Queens College Department of Biology, Queens, New York, USA

**Keywords:** *Mycobacterium tuberculosis*, lineage 1, complete genome

## Abstract

*Mycobacterium tuberculosis* (MTB) presents a global health issue. Various genotypes of MTB have different geographic distributions. The majority of MTB in Thailand belong to lineages 1 and 2. Here, we report the complete genomic sequences of MTB sublineage L1.1.1.7, which is common in Thailand and infrequently identified in other countries.

## ANNOUNCEMENT

*Mycobacterium tuberculosis* (MTB) in Thailand is genetically diverse with the majority belonging to lineage 1 (L1, Indo-oceanic or East-African-Indian strains) and L2 (East Asian strains) (1). Three L1 sublineages, L1.2.2.2, L1.1.1.7, and L1.1.1.8, were particularly common in Thailand, contributing to approximately 44% of L1 (2). L1.1.1.7 was reported infrequently in India, Bangladesh, and Vietnam (3, 4). We investigate the complete genomes of two L1.1.1.7 isolates.

MTBCR170031 and MTBCR170913 were isolated from the sputum of pulmonary TB male patients in Chiangrai in 2017 and 2020, respectively, approved by the Ethical Committee of Chiangraiprachanukroh Hospital. Both were isolated in Lowenstein-Jensen medium, identified by BD MGIT TBc Identification Test and tested sensitive to isoniazid and rifampin using a fluorometric method (BACTEC MGIT960, Becton-Dickinson, USA) (5). DNA of both isolates was extracted using Presto Mini gDNA Bacteria Kit (Geneaid Biotech, Taiwan) and sequenced using Illumina NextSeq 500 after their 150-base read library was prepared using the NexteraXT DNA Library Preparation Kit (Illumina, USA). The short read data were quality controlled using the Trimmomatic v0.32, ensuring an average read quality of over 30 and a minimum read length more than 60 bp (6). A library of MTBCR170913 was prepared from 300 ng of unsheared DNA and sequenced by MinION Flow Cell R10.4 with Kit 12 chemistry; SQK-NBD112.24 ligation-based sequencing kit. Porechop v0.2.4 was used to trim adapters and threads set to 16. The data were quality controlled by Filtlong v0.2.1 with the minimum mean quality more than 0.8 and minimum read length more than 1,000. Base calling was done by Guppy (7). A 20 kb library of MTBCR170031 was constructed for P6-C4 chemistry from sheared DNA without size selection and sequenced on the RSII platform (Pacific Biosciences, Menlo Park, USA). The data quality control was done similar to Nanopore data. The hybrid assembly and circularization of both set of data were done using FlyE v2.9.2 (8) with genome size setting of 4.4 million bases. Error correction was done by Pilon v1.24 (9) and presented using QUAST v5.2.0 (10). Default parameters were used except noted otherwise. The genomes were rotated manually to match the start base with the H37Rv genome.

The hybrid assembly of each sample formed a single circular chromosome without any plasmids as summarized in Table 1. Dot plots against the H37Rv genome revealed no major genome rearrangement (data not shown). The assembled sequences were annotated with PGAP at NCBI. Drug resistance mutations were identified using TB-Profiler (11), which revealed only a mutation at position 1381 of *gyrB* of MTBCR170031. The CRISPR regions of MTBCR170031 and MTBCR170913 were 11111111111111111111111111111111100000000000000000000011111111111111 and 11111111111111111111111111111111111111000010111000000000001111111111 (12), where 1 and 0 represent the presence and absence of a DVR, resulting in the spoligotypes of SIT1274 (777777760000071) and SIT934 (777777777413071), respectively (13). The deletion of DVR34-54, including IS*6110* between DVR34 and DVR35, found in MTBCR170031, is common among L1.1.1.7 (2). There were three IS*6110* elements in MTBCR170031 and five in MTBCR170913, with only one shared by both.

**TABLE 1:** Information regarding both samples of L1.1.1.7 from northern Thailand^*a*^

Sample ID	MTBCR170031	MTBCR170913
Ages of patients (years)	71	63
Sample collection dates	2017–01-23	2020–03-20
Short read Accession numbers	SRR28776755	ERR12328191
Numbers of reads	363,018	1,782,970
Short read depths (X)	19.4	116.7
Short read coverages (%)	99.66	99.99
Long-read sequencing technology	PacBio	Minion Nanopore
Long read Accession numbers	SRR28788881	ERR12328567
Numbers of reads	177,373	48,949
Raw read N50 (bp)	9,138	3,661
Complete genome accession number	NZ_CP097109.1	CP152406.1
Genome lengths (bp)	4,417,031	4,416,753
% GC content	65.5	65.6
Numbers of genes	4128	4134
Numbers of pseudogenes	224	288
Spoligotypes	777777760000071	777777777413071
SIT	1274	934
24-loci VNTR type^*a*^ (14)	2247**4**42**4**2**17**32**4**5223354**4**13	2247**3**42**5**2**28**32**2**5223354**6**13
Number of copies of IS*6110*	3	5

^*a*^ The bold numbers indicate the different copy numbers between both isolates.

This work consolidates scarce resources of L1 complete genomes, aiding further comparative genomic studies.

## Data Availability

The genome sequence data project has been deposited in GenBank under the accession numbers shown in Table 1.
